# Recurrence of Congenital Diaphragmatic Hernia: Risk Factors, Management, and Future Perspectives

**DOI:** 10.3389/fped.2022.823180

**Published:** 2022-02-09

**Authors:** Francesco Macchini, Genny Raffaeli, Ilaria Amodeo, Martina Ichino, José Luis Encinas, Leopoldo Martinez, Lucas Wessel, Giacomo Cavallaro

**Affiliations:** ^1^Department of Pediatric Surgery, Fondazione IRCCS Ca' Granda Ospedale Maggiore Policlinico, Milan, Italy; ^2^Neonatal Intensive Care Unit, Fondazione IRCCS Ca' Granda Ospedale Maggiore Policlinico, Milan, Italy; ^3^Department of Clinical Sciences and Community Health, Università degli Studi di Milano, Milan, Italy; ^4^Department of Pediatric Surgery, La Paz Children's Hospital, Universidad Autónoma de Madrid, Madrid, Spain; ^5^Department of Pediatric Surgery, Faculty of Medicine Mannheim at Heidelberg University, Mannheim University Medical Center, Mannheim, Germany

**Keywords:** congenital diaphragmatic hernia, hernia recurrence, minimally invasive surgery, pulmonary hypertension, mortality, prosthetic patch, FETO

## Abstract

Recurrence is one of the most common surgical complications in Congenital Diaphragmatic Hernia (CDH). It could remain clinically silent for a long time or present as an acute complication week, months, or even years after the primary surgery. Several risk factors have been identified so far. An extended diaphragmatic defect represents one of the leading independent risk factors, together with indirect signs of large defect such as the liver position related to the diaphragm and the use of the prosthetic patch and with the use of a minimally invasive surgical (MIS) approach. However, the exact contribution of each factor and the overall risk of recurrence during the life span still need to be fully understood. This mini-review aims to give an overview of the current knowledge regarding CDH recurrence, focusing on predisposing factors, clinical presentation, management and follow-up of high-risk patients, and future perspectives.

## Introduction

Recurrence of Congenital Diaphragmatic Hernia (CDH) represents a common complication in CDH survivors, along with pulmonary, gastrointestinal, neurobehavioral, and developmental anomalies (1–4). It mostly happens at the site of the original hernia, but occasionally hiatal hernia may follow CDH repair due to tension on the diaphragmatic crura. Therefore, we will concentrate on this review over the first entity.

The incidence of recurrence after CDH repair varies considerably, ranging from 5 to 65% in reports with different lengths of follow-up and different follow-up protocols (4–11). The average age at recurrence is 12 months, with 47.6% of cases occurring before 1 year of age, 76.2% before 2 years, and near 100% before 5 years (12–14). Only 3% of cases are reported as an early in-hospital recurrence (2). In older children, the recurrences are rare (15).

## Predisposing Factors

Many different predisposing factors (PF) have been investigated related to pre- and postnatal life, congenital and acquired diseases, medical and surgical problems, with inconclusive results in different series.

### Prenatal

Although there are authors that did not evidence differences in recurrence rate among prenatal patient-related characteristics (16), most studies report a higher recurrence rate in patients with signs likely related to a larger defect size such as lower observed/expected lung to head ratio (O/E LHR%), prenatal diagnosis of CDH (<22 weeks of gestational age), observed/expected total fetal lung volume (O/E TFLV) <30%, thoracic position of the liver (5, 8, 10, 17, 18). A recent study by Amodeo et al. showed that patients prone to recurrence have lower final O/E LHR% during fetal life and could be identified in the early postnatal life by estimating the pulmonary surface at the first Chest X-ray (CXR) control after birth. Indeed, the unit increase in total and ipsilateral area in cm^2^ was associated with a 14 and 29% reduction in the risk of recurrence, respectively (17). These findings further suggest that recurrence is related to the defect size. In addition, a large defect size has been associated with an early in-hospital recurrence (2). Another prenatal risk factor frequently reported in the literature is the absence of a hernia sac (5, 10, 18–20). There is still contrasting evidence concerning a higher recurrence risk in the right-sided defects (21, 22), while Fetal Endoscopic Tracheal Occlusion (FETO) procedure has not been confirmed as a predisposing factor for recurrence (2, 17, 21).

### Postnatal

Many postnatal PFs seem to be associated with recurrence. Some might be indirect signs of larger defects, such as the need for ECMO and the use of diaphragmatic and abdominal patches. Others are generally related to the severity of the disease, such as prolonged invasive respiratory support, need for intensive care, longer duration of mechanical ventilation, post-operative sildenafil requirement, longer length of stay (LOS), age at discharge, supplemental oxygen requirement, persistent pulmonary hypertension. And others still, like thoracotomy and MIS, are related to surgical choice (12, 17, 23–25).

**Figure 1:**
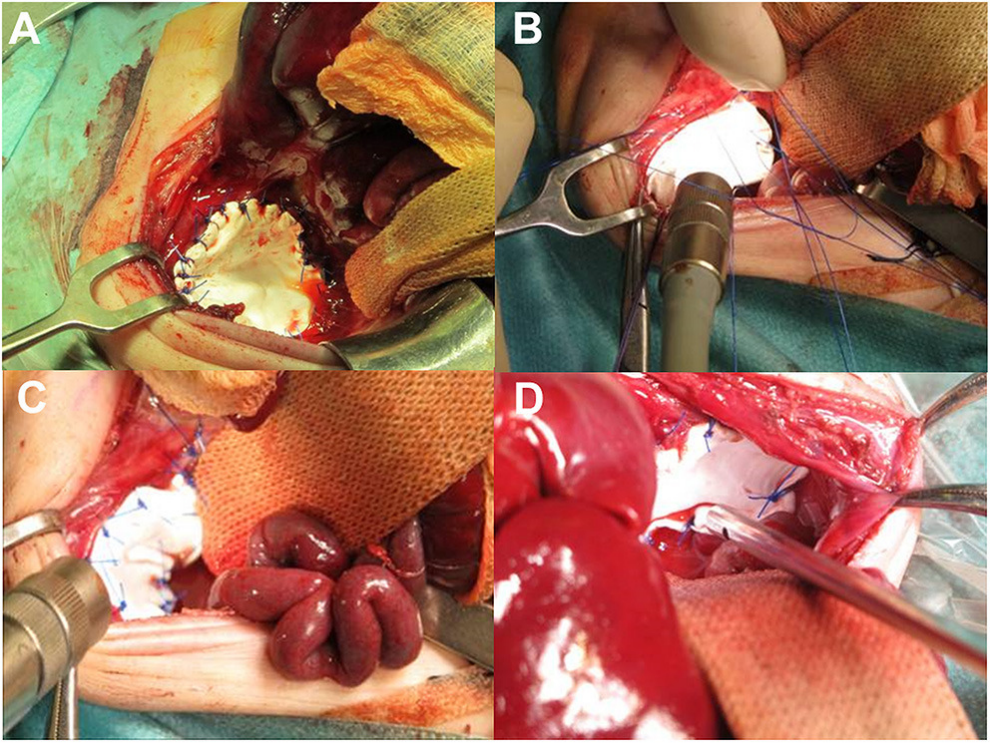
Intraoperative imaging of patch repair. **(A,B)** Dome-shape patch repair. **(C,D)** Cone-shape patch repair.

Surgical-related PFs seem to have a major role in recurrence among postnatal variables, especially the use of patches, both diaphragmatic and abdominal (16). Despite this, the use of patches for repair has been increasing in the last decade. Patients who require a diaphragmatic patch repair are reported to have a risk 2.83 times higher of developing a recurrence (26). The inability of the synthetic patch to grow with the patient is the mechanism underlying this strong association (2). But, again, the disease's severity and the defect's size may present an underlying independent role (26). The goal of the patch is to allow closure of the defect without tension on the surrounding structures, despite a large defect size, granting a tension-free suture. This aims to reduce the risk of recurrence and seems effective, as shown by Zahn et al. (27). Another advantage is the possibility to create an “over-sized” cone- or dome-shape for the new diaphragm, allowing for better respiratory physiology. A cone- or dome-shaped prosthetic patch gives the thoracic cavity a more physiologic shape and volume (Figure 1). Moreover, it provides additional abdominal volume during the significant growth of the first year of life, facilitating tissue ingrowth coming from folds of the redundant material sutured to the rims of the diaphragm (28). Nonetheless, some single-center studies do not report any significant difference in hernia recurrence rates between the patch and primary repair, while other authors even described a reduced recurrence rate in patients treated with a patch (6, 27, 29, 30), in contrast with data of large series from high-volume centers (6, 16, 26).

Another open issue is the patch material. In a recent report, the non-absorbable polytetrafluoroethylene (PTFE) patch appears to have a lower recurrence rate than the absorbable intestinal submucosal (SIS) patch. This retrospective and monocentric study assessed the use of patches with a follow-up limited by the bias due to the sequentially timed implementation of the PTFE patch related to the SIS patch (31). Albeit the article by Camila et al. presents some limitations, the future seems promising for using PTFE patches (31).

Alternative methods for diaphragmatic breach closure have been suggested to avoid diaphragmatic patches, such as wall muscle flaps like the reversed latissimus dorsi muscle flap. This is suggested as an alternative to patches in case of significant defects or agenesis of the hemidiaphragm (28). Limited experiences have shown similar or even better outcomes with muscle flaps (32, 33). However, larger studies would be needed to confirm these results, and strong evidence in its favor is still failing. Moreover, the problem in muscle flaps is that innervation is missing, and we could see a marked dysfunction of diaphragm motility overall in massive C- and D-defects (34, 35).

Constant efforts are being made to find the “perfect” graft for diaphragmatic reconstruction, and the future of tissue-engineered diaphragmatic repair is promising (36).

Based on current evidence, major international study groups recommend using non-absorbable prosthetic patches, mainly PTFE, aiming at an oversized/dome shape. PTFE appears safe and is associated with a low recurrence rate (7, 10, 26, 37).

Another surgical PF is the use of an abdominal patch. Even if rapidly removed through staged closure, an abdominal prosthesis can predispose to recurrence by interfering with the integrity of the diaphragm at its connection to the anterior abdominal wall (16, 27).

Most surgeons agree that the recurrence rate also depends on the surgical technique (28). The postero-lateral section of the defect deserves particular attention and is deemed essential to secure the patch with particular care in this part of the defect, passing the stitches around the ribs and intercostal muscles, if necessary. Usually, a non-resorbable suture is used to secure the diaphragmatic patch (28). In addition, some technical expedients have been proposed to minimize the risk of recurrence. For instance, pledged sutures are used to strengthen the hold on the tissue or to tailor the patches in modified shapes such as double-layer patches (18).

The post-operative chest X-ray (CXR) may help evaluate the accuracy of surgery, and a flat-appearing diaphragm could be an indirect sign of a tense repair with a higher risk of recurrence. However, no relationship between post-operative CXR diaphragmatic appearance and recurrence has been observed (38).

Recently, minimally invasive surgery (MIS) has increased its pediatric and neonatal surgery applications, but CDH still represents a challenge for laparoscopic (anterior defects) and thoracoscopic (mostly Bochdalek defect) repairs. The advantages of MIS are mainly represented by less pain, less incisional complications, and reduced surgical stress compared to traditional surgery. In general, TR is not contraindicated in newborns since relative hypercapnia is tolerated (29). At the same time, thoracoscopic repair (TR) of CDH is reported to have a greater risk of recurrence (2–9%) than the classic repair through laparotomy (1–4%) (2). Cioci et al. also observed a significantly higher recurrence in those patients who underwent MIS repair (48%) as compared to open repair (OR) (16%) (23). However, significance was not reached in other series (30), and some recent studies have identified a similar risk of recurrence between TR and OR in selected patients (39–45).

Furthermore, the rate of recurrence in TR decreases with the increase of the surgeon's experience (learning curve) (2). Because the increased risk of recurrence with MIS repair would seem due to surgeon inexperience, several studies proposed that TR should be limited to high-volume centers and experienced surgeons (2). Nevertheless, other factors could be involved in the higher recurrence risk in MIS. Therefore, it has been suggested to limit MIS to the smallest defects, classified as A or B, by the Congenital Diaphragmatic Hernia Study Group (CDHSG) Staging System (23). Additional proposed selection criteria are cardiovascular stability and no pulmonary hypertension, mild symptoms or asymptomatic, liver down, late presentation or postnatal diagnosis, and absence of severe comorbidities (31, 46). However, further studies are needed, especially with a structured long-term follow-up.

At present, no correlation has been reported between time to surgery and risk of recurrence (16). The correlation between ECMO support and recurrence also requires some attention. The need for ECMO could independently increase the risk of recurrence or indicate a more severe clinical presentation with a larger defect size (2). Moreover, the recurrence rate is not associated with the repair timing (before, during, or after ECMO) and the need for the “EXIT to ECMO” procedure (2, 47, 48). However, these results are biased by the lack of standardized long-term follow-up in some series (2, 49).

A recent study observed the impact of hospital volume on CDH recurrence for the first time. Cioci et al. demonstrated as low-volume CDH centers have significantly higher recurrence rates and hospital costs than high-volume CDH hospitals. Therefore, the de-centralization of CDH patients would be a further PF (23). Consequently, through a hub and spoke model, the centralization of CDH delivery is needed to improve care and reduce costs, complications, morbidity, and mortality (50).

## Management

The management is based on the severity of the condition. A minor recurrence is defined as a tiny defect in an asymptomatic patient, with minimal herniation of the abdominal content into the thorax, more frequently only the omentum, without worsening during follow-up. Recurrence is defined as major when it allows the stomach and/or bowel loops to re-herniate back up into the thorax or worsens over time (5, 6, 15).

In case of minor recurrences, conservative management may represent a good choice, avoiding re-operation, provided that the patient remains stable at periodic plain CXR and clinical examination for a minimum follow-up of 5 years (5, 6, 15, 27).

**Figure 2:**
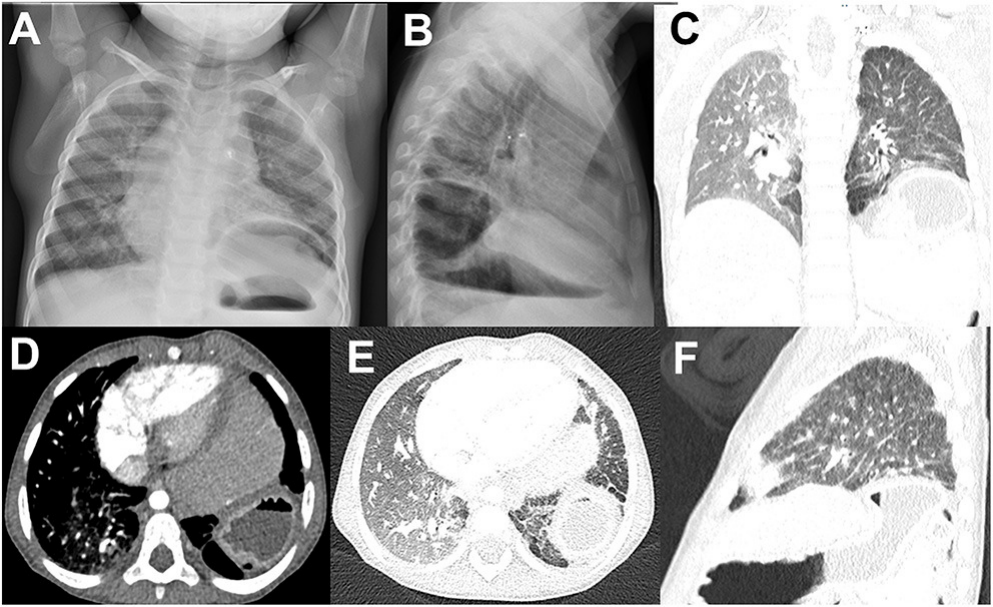
Radiological image of the major recurrence of left side CDH after a first patched diaphragmatic closure. **(A,B)** Chest X-ray image, **(C–F)** computed tomography image.

A surgical approach is indicated when a major recurrence is detected (Figure 2). At the dorsal costo-abdominal place, the sutures could grow through the ribs or could be torn out, leading to relatively small additional defects in the case of Bochdalek hernia. Nevertheless, a fault at the hiatus could be observed in other patients. Therefore, sometimes, an additional patch could be inserted without replacing the entire patch in both cases. Thoracotomy could be a good alternative in cases where the recurrence was located more ventral. Despite this, adhesions could even affect the thoracic cavity.

There is ultimately no consensus on the optimal surgical approach to CDH recurrence. Some authors suggest approaching a recurrence from a so-called “virgin plane”, meaning the opposite body cavity compared to the first surgery (51). This aims to work in a more accessible surgical field with fewer adhesions and better visibility.

However, a recent survey shows that recurrence is repaired with the same technique (laparotomy, thoracotomy, MIS) as the primary operation in 48% of cases (23). In the open approach, laparotomy is always favored over thoracotomy. On the other hand, thoracoscopy is the preferred approach among MIS surgeons for the first surgery as well as recurrence, except in case of initial thoracotomy. Future prospective studies may help define the optimal approach. Still, in the absence of clear evidence favoring a specific technique, it is preferable to use the most comfortable route to the operator.

## Re-recurrence

The incidence of a second recurrence after the first recurrence repair is not well documented in the literature, but it seems to be high, especially in D-defects. Moss et al. reported a second recurrence rate of 25% (52). In another series by Laituri et al., the frequency of a second recurrence was 50% among patients with CDH repaired with the patch (53). Another study showed a second recurrence rate of 19%, and re-repair was performed either by patch or by primary suturing (12).

Considering the risk of subsequent recurrences, a long-term multidisciplinary follow-up plays a key role in the timely detection of complications. Because re-re-operations are very demanding, a subtle technique in order to avoid further complications is needed. The previous patch can be left inside and a second patch added over it to reduce the risk of iatrogenic damage.

## Clinical Presentation and Follow-Up for Timely Diagnosis of Hernia Recurrence

Clinical presentation of CDH recurrence may include dysphagia, retching, constipation, abdominal pain, failure to thrive, and progressive dyspnea up to respiratory failure. However, upper gastrointestinal symptoms should be carefully assessed to differentiate between reflux disease and possible hiatal hernia from recurrence. Sometimes, an acute bowel obstruction could be the presenting clinical picture of a misdiagnosed hernia recurrence. However, up to two-thirds of the patients are asymptomatic at the detection, and its diagnosis remains extremely difficult when no structured follow-up is offered (5, 27).

Considering the high overall recurrence rate and the insidious clinical presentation, multidisciplinary management and follow-up of CDH patients are recommended, and it is advisable to consider specific follow-up algorithms depending on the patient's risk of recurrence (11). However, it is unclear if active searching with periodic imaging is warranted in all patients for timely recognition of the complication since unnecessary radiation could be avoided in those with low recurrence risk (16).

Since recurrence could occur at any time during the years following primary repair, it would be helpful to promote a remote follow-up that includes a multidisciplinary team of neonatologists, pediatrics, and pediatric surgeons at 3, 6, 12, 18, 24 months of life and then annually until the age of 8 years (12, 47). In addition, standardization of clinical and radiological assessments should be implemented, even for asymptomatic patients (5, 47). CXR should be scheduled at 12 and 24 months and performed anytime as needed, based on the patient's clinical symptoms. Then, it should be planned every 2 years until 8 years old for primary closure, with an additional 18-month CXR for patients undergoing patch repair (47). The preferred diagnostic exams to detect a CDH recurrence are the upper gastrointestinal (UGI) contrast study, barium enema, and computed tomography (CT) scan (54).

## Conclusions and Future Perspectives

There is a diffuse agreement that a tension-free diaphragmatic repair with the use of a cone/dome-shaped patch is advisable to reduce the risk of hernia recurrence during the patient's growth (29). However, no specific data definitively show the superiority of biological or synthetic patches (32, 53, 55, 56). The PTFE appears to be associated with a low recurrence rate and is recommended by international groups (10, 57). However, it would be helpful to perform randomized control trials to demonstrate its superiority over absorbable patches (7, 31).

Tissue engineering seems to be the final answer to the search for a perfect diaphragmatic replacement, but many issues still need to be addressed to optimize these techniques for clinical practice (36).

A careful imaging evaluation before patients' discharge is necessary, especially when relevant risk factors for recurrence are present, such as MIS or extensive defect repair (2).

Ultimately, the centralization of CDH patients to referral high-volume centers is pivotal to manage possible complications with an appropriate and customized follow-up plan (23).

## Author Contributions

FM, GC, GR, IA, MI, and LM contributed to the study's conception and design. FM, GR, MI, IA, and GC wrote the first draft of the manuscript. FM and GR contributed equally and had the right to list their names first in their Curriculum Vitae. IA, JE, LM, and LW provided extensive critical revision. All authors contributed to the manuscript's critical revision, read and approved the submitted version.

## Conflict of Interest

The authors declare that the research was conducted in the absence of any commercial or financial relationships that could be construed as a potential conflict of interest.

## Publisher's Note

All claims expressed in this article are solely those of the authors and do not necessarily represent those of their affiliated organizations, or those of the publisher, the editors and the reviewers. Any product that may be evaluated in this article, or claim that may be made by its manufacturer, is not guaranteed or endorsed by the publisher.
